# Impact of Insurance Type on Access to Pain Management Specialists for the Treatment of Lower Back Pain

**DOI:** 10.7759/cureus.51668

**Published:** 2024-01-04

**Authors:** Phillip Cifuentes, Manuela Jaramillo, Fabio Garrote, Daniel Bravo, Juan C Alvarez, Ramon M Quintero, Stephan Mouhanna, Rakesh R Nair

**Affiliations:** 1 Anesthesiology, Florida International University, Herbert Wertheim College of Medicine, Miami, USA; 2 Internal Medicine, Albert Einstein College of Medicine, New York City, USA; 3 Dr. Kiran C. Patel College of Osteopathic Medicine, Nova Southeastern University, Fort Lauderdale, USA; 4 Anesthesiology, Memorial Regional Hospital, Hollywood, USA; 5 Cellular Biology & Pharmacology, Florida International University, Herbert Wertheim College of Medicine, Miami, USA

**Keywords:** chronic pain management, healthcare inequality, access to health care, chronic low back pain (clbp), medicare patients

## Abstract

Background

Low back pain is known to be one of the leading causes of disability among the young and elderly population. Low back pain can stem from multiple sources, including spinal degeneration, injury, herniated discs, sciatica, and other contributing causes. This symptom significantly influences the quality of life of affected individuals. Its implications include extensive social and economic costs. Economic considerations arise from the fact that not all healthcare facilities accept the insurance plans available to retired individuals under Medicare. This places an additional burden on patients who must bear the financial responsibility for healthcare services not covered by their insurance plan. Florida, renowned as a favored state for retirement, consists of a demographic composition wherein 21% of its residents are aged 65 or older. A significant proportion of this demographic qualifies for Traditional Medicare (TM) and/or Medicare Advantage (MA) plans. Thus, understanding the disparities in healthcare access between Medicare and Medicare Advantage plans is crucial. This study aims to evaluate different Medicare insurances available in the market and their impact on the ease of accessibility to pain management specialists for the treatment of lower back pain in Florida patients.

Methods

We analyzed the Florida Department of Health database to identify the four counties in Florida with the highest Medicare enrollment rates in 2022: Miami-Dade, Palm Beach, Broward, and Pinellas County. Using the U.S. News and Report directory, 25 Pain Management-trained anesthesiologists were randomly selected from each of the four counties. Each office was contacted four times via telephone by four different team members to assess appointment availability for a fictional 65-year-old grandfather seeking treatment for chronic low back pain. The study examined appointment availability and accepted insurance types, including Cigna (commercial insurance), TM, Humana Gold Plus HMO (Medicare Advantage plan), and Blue Medicare Select PPO (Medicare Advantage plan). Practices without contact information or retired physicians were excluded from the analysis. Time to appointment was measured in business days.

Results

Of the 100 Pain Management Physicians contacted, 44 fit the inclusion criteria of being non-retired physicians, still practicing in one of the four counties with open offices and valid contact information. Blue Medicare Select PPO was accepted by 47.73%, Humana Gold Plus HMO by 56.82%, TM by 93.18%, and Cigna by 93.18% of the encounters. Blue Medicare select PPO and Humana Gold Plus HMO were accepted at significantly lower rates when compared to Traditional Medicare and Cigna with P values of P < .00001 and P < .000176, respectively. There was no significant difference found in the time to appointment between insurances with P value < 7.

Conclusion

The study found that patients enrolled in Medicare Advantage plans have significantly decreased access to care when compared to those enrolled in TM or commercial insurance. Further research is needed to elucidate the reasons behind differences in access to care across different insurances, as identified in the study.

## Introduction

More than 17 million older adults suffer from one episode of lower back pain annually, and it is one of the most common reasons patients seek healthcare services [1]. In 2020, lower back pain affected approximately 619 million people globally with a projected 843 million cases by 2050 [2]. Rates of lower back pain increase with age, with the highest proportion of lower back pain occurring between ages 50 and 60 and then stabilizing after the seventh decade of life [3]. Along with the increased prevalence of low back pain, there has been an increase in low back pain documentation rates in Medicare beneficiaries [4].

Representing 22 million, approximately 34% of Medicare beneficiaries are enrolled in a Medicare Advantage plan [5]. Historically, the Traditional Medicare (TM) system has functioned through a fee-for-service reimbursement model. On the other hand, the Medicare Advantage (MA) program, also known as Medicare Part C, is composed of private plans that are funded through capitated government payments. Through capitated payments, healthcare providers or organizations receive predictable upfront payments to cover a patient's healthcare cost over a certain amount of time. These private insurance plans utilize other aspects of managed care, such as healthcare networks, utilization reviews, and adjustable contracts with healthcare professionals and organizations that provide alternatives to fee-for-service reimbursement [6].

Though Landon et al. found that while MA was associated with a decreased 30-day mortality in 2009, these differences in mortality and almost all quality measures between TM and MA were erased by 2018 [7]. Further analysis of these Medicare Advantage plans has revealed various factors that might hinder the quality of care for Medicare Advantage patients. In most Medicare Advantage plans, the networks of physicians are limited. When compared to Traditional Medicare recipients, MA beneficiaries were found to receive care from lower-quality hospitals, nursing homes, and home health providers [8]. These studies also note that MA beneficiaries who have greater health needs have higher rates of disenrollment. Additionally, it has also been noted that MA beneficiaries may encounter elevated rates of prior authorization, potentially resulting in delays and impediments to care due to the intricate nature of paperwork and insurance companies possibly deeming the procedure unnecessary [8]. All of these factors can lead to a delay in access to care. Though studies investigating the differences in access and quality of care for TM and MA beneficiaries have been unable to reach a consensus on the superiority of either insurance model, there has been limited research on differences between the access to pain management care.

Due to the high prevalence of lower back pain in the elderly population, the majority of whom are enrolled in Medicare or Medicare Advantage plans, it is imperative to understand how TM to MA coverage influences access to chronic lower back pain care. The purpose of this study is to evaluate whether enrollment in a Medicare Advantage plan affects access to pain management specialists for the treatment of lower back pain. Typically, after attempts to control symptoms of lower back pain with primary doctors, patients are commonly referred to fellowship-trained pain management specialists. These specialists employ a multidisciplinary approach to assess and manage low back pain, combining a range of modalities such as medication management, interventional procedures, physical therapy, and rehabilitation. We hypothesize that beneficiaries of Medicare Advantage plans will have reduced access to pain management specialists for the treatment of lower back pain when compared to those with Traditional Medicare or commercial insurance.

## Materials and methods

The U.S News and Report directory was used to accumulate a short list of Pain Management-trained anesthesiologists. To mitigate bias, physicians excluded from the analysis were Pain Management specialists who were not anesthesiologists, as well as practices without contact information and retired physicians. The Florida Department of Health database was used to identify the top four counties in the state of Florida with the highest Medicare enrollment rates: Miami-Dade, Palm Beach, Broward, and Pinellas County. In total, we identified 25 Pain Management-trained Anesthesiologists from each respective county. To ensure the information collected was accurate, an internet search was performed to confirm the current information for each office. Identified physicians were then randomly assigned a serial number of 1-100.

Insurance comparisons were planned between three categories, 1. traditional insurance 2. commercial insurance, and 3. MA plans. Humana Gold Plus (HMO) and Blue Medicare Select (PPO) were selected due to their closely aligned enrollment rates covering 5.5 and 4.4 million, respectively, rendering them the most comparable among all MA plans. Cigna, chosen as the commercial insurance representative, holds a position among Florida's top three private insurers. To prevent voice recognition during calls from introducing bias into the data, a systematic approach was employed by the research team. One team researcher initiated contact with the clinic inquiring about a specific type of insurance. Subsequently, another team researcher contacted the clinic independently inquiring about a different type of insurance. This process was done for all four insurance types. During normal business hours (9 am - 4 pm), each clinic was called once per insurance. To simulate a real-life scenario, each researcher used a script (Appendices) and called on behalf of their fictional grandfather above the age of 65 who was seeking treatment for chronic low back pain. Callers then recorded whether or not the insurance was accepted, the number of business days till the earliest appointment, any reasons the patient could not be seen, and any issues during the call such as the office being closed for lunch or a call sent straight to voicemail. Researchers recorded these responses on a premade data collection sheet in Microsoft Excel (Microsoft Corporation, Redmond, WA).

Callers were given instructions for situations where it was difficult to make contact with an office. If callers were sent to voicemail or placed on hold for an extended period, they called back at a later date. Twenty-five clinics that were called on four separate occasions without success were eliminated from the study. Thirty-one clinics were eliminated either because the clinic closed down, the physician had retired or moved to another state. Forty-four clinics were ultimately included in the study.

The calls were conducted during the months of March and April. This time frame was useful to account for potential differences in availability due to holidays. Furthermore, to avoid withdrawing resources for real-world patients, callers were instructed to never book or confirm an appointment time slot.

This study, like other “secret-shopper” studies, did not involve the use of human subjects or patient records. For this reason, informed consent was not required. The study solely relied on organizational data; Institutional Review Board approval was not needed.

## Results

**Figure 1:**
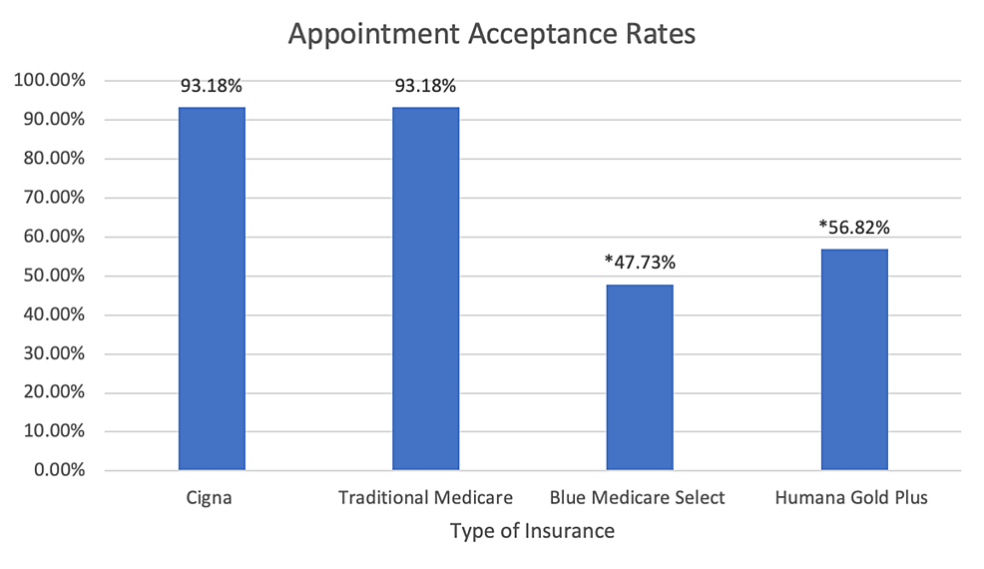
Illustration showing the rate of acceptance for each insurance type * Indicates statistical significance when compared to Traditional Medicare and Cigna

Of all the offices contacted, 47.73% of them accepted Blue Medicare Select PPO, Humana Gold Plus HMO at 56.82%, Traditional Medicare at 93.18%, and Cigna at 93.18% (Figure 1). When compared to both Traditional Medicare and Cigna commercial insurance, Blue Medicare select PPO and Humana Gold Plus HMO were accepted at significantly lower rates with P values of P < .00001 and P < .000176, respectively. Moreover, acceptance rates between Traditional Medicare and Cigna were not statistically significant with a P value of <1. Comparing acceptance rates between Blue Medicare Select PPO and Humana Gold Plus HMO showed no statistical significance with a P value of < .2853.

**Figure 2:**
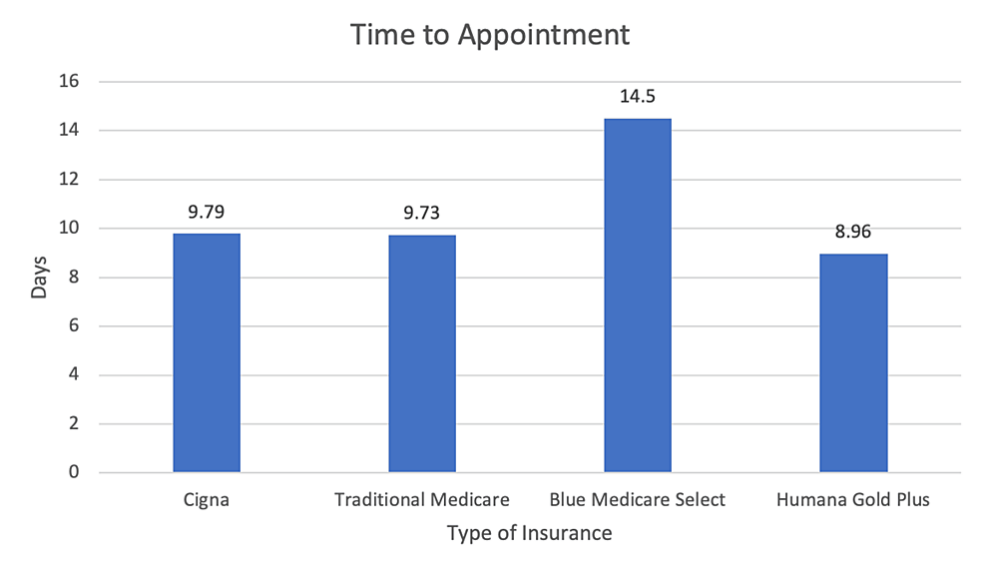
Graph demonstrating business days until the first available appointment for each insurance type

In terms of average time to appointment in business days, Blue Medicare Select PPO had 14.50 (SD 3.54), Humana Gold Plus HMO had 8.96 (SD 8.65), Traditional Medicare had 9.73 (SD 9.62), and Cigna had 9.79 (SD 5.52) (Figure 2). There was no significant difference found in the time to appointment between insurances with P value < 0.694032.

## Discussion

In this study, we adopted a secret shopper methodology to investigate patient access to anesthesia-trained pain management specialists for lower back pain across different insurance types in Florida. While this methodology has been used in healthcare research, it is notably the first study, to our knowledge, to assess how Medicare Advantage (MA) enrollees access pain management specialists for lower back pain [9-11]. In response to the elevated incidence of lower back pain within the elderly population, our decision to investigate patient access to chronic lower back pain specialists is grounded in our commitment to understanding this prevalent health concern [12]. The state of Florida was chosen due to its notable demographic composition, with the second-highest concentration of elderly residents in the United States, the majority of whom are enrolled in Medicare or Medicare Advantage plans [13]. Given the expanding elderly population and the growing incidence of lower back pain, it is essential to gain insights into the impact Medicare Advantage coverage has on access to lower back pain management [14,15].

Our study found that Traditional Medicare (TM) and commercial insurance (Cigna) had considerably higher acceptance rates than MA plans, both Humana Gold Plus HMO and Blue Medicare Select PPO. This finding indicates that patients enrolled in MA plans face difficulty obtaining appointments for lower back pain care [8]. While some have speculated that MA patients may underutilize services due to a healthier starting cohort or an increased focus on preventative care in MA plans, our results suggest that a decreased ability to access care might be a significant contributing factor [16].

Decreased access to healthcare may decrease the quality of life of patients suffering from lower back pain. Chronic lower back pain has been found to have been significantly associated with disability. Disability of any kind has a significant psychological impact on patients [16]. A 2007 study in Iranian patients with lumbar disc herniation found that functional disability and pain correlate with scores on both anxiety and depression [17]. Studies have also demonstrated that there have been significant correlations between chronic pain and persistent states of fear, anxiety, and depression [18,19]. Delays in access to treatment could lead to further negative implications for the elderly population suffering from lower back pain. Therefore, initiatives aimed at enhancing transparency within the health insurance sector, encompassing factors like insurance acceptance rates and coverage details may help consumers make optimal health insurance decisions that could in turn increase access to care. This transparency has the potential to foster improved patient health outcomes and overall quality of life.

The requirement for referrals may contribute to variances in appointment success rates, as evident in many MA plans, potentially impeding access to or delaying the provision of pain management care. King et al. demonstrated how increased access to referrals significantly reduced delays in patients being seen in specialist macular clinics compared to traditional General Practitioner-centered referral systems [20]. Another factor that could explain the differences in appointment success rates may be reimbursement rates. Some studies have shown that MA plans may reimburse healthcare providers at rates lower than TM or commercial insurance. While we did not have access to reimbursement rate data in our study, this remains a plausible explanation for the disparities in appointment success rates [21].

Limited access to pain management services for MA enrollees could potentially exacerbate economic and racial disparities [22]. Racial minorities and low-income patients, who are disproportionately covered under MA, may already face challenges in accessing care [14]. Our findings suggest that these patients may also be experiencing decreased access to Pain Management services. Furthermore, MA plans typically have more limited provider networks to control costs. In a cross-sectional analysis of cumulative patterns in disenrollment rates among Medicare Advantage beneficiaries, it was observed that 48% of non-dually enrolled individuals and 53% of dually enrolled beneficiaries terminated their contracts within a five-year period [23]. Furthermore, the study identified higher disenrollment rates among Black beneficiaries, as well as among those with pronounced health needs and greater comorbidity burdens [23]. These findings hold significance, as health plans might pursue financial benefits by increasing coding practices over a brief duration while potentially circumventing interventions aimed at addressing chronic conditions, such as lower back pain.

An important aspect highlighting the consequences of postponing healthcare is the financial challenges faced by elderly individuals without insurance coverage. Given that TM and MA plans offer more affordable avenues for healthcare coverage, it is not uncommon for individuals in this demographic to delay enrollment until the age of 65, inadvertently neglecting their healthcare needs. The delayed treatment of conditions such as back pain carries potential consequences that negatively impact the quality of life and overall physical and mental health [18]. Being part of an insurance model that restricts access to healthcare providers could further compound the delay in care. Our study identified a discrepancy in acceptance rates between TM and MA plans. Potential solutions to enhance access to care involve increasing transparency in Medicare insurance coverages and acceptance rates for beneficiaries. This approach enables individuals to make more informed decisions and select a plan that provides greater access to physicians in their area, thereby improving overall access to care.

Limitations

We should consider some essential points regarding the limitations of this study. First, our approach involved using the "secret shopper" methodology, which, while common in healthcare research, might not give a complete picture of what real patients experience. The responses we received during phone calls might only partially reflect appointment availability or the quality of care. Second, this study focused on four specific counties in Florida, and these areas may not represent the broader population. Our findings may not apply to other areas with different healthcare systems. Only 44% of the physicians met the initial inclusion criteria, which limited the significance of our findings. Anesthesiologists were randomly picked and some physicians were simply not within the provider networks of MA plans affecting insurance acceptance rates. In addition, only two medicare advantage plans were selected which might affect results as there are many more MA plans available. Despite these limitations, our research sheds light on the differences in insurance acceptance rates between TM and MA beneficiaries seeking chronic Pain Management specialists for lower back pain treatment.

## Conclusions

Our study highlights disparities in access to anesthesia-trained pain management specialists for chronic lower back pain between traditional Medicare and Medicare Advantage Enrollees. With MA enrollees experiencing reduced opportunities for care, future research should explore the drivers of these disparities and seek to identify potential solutions. These inequalities have wide-reaching implications for healthcare accessibility, quality, and equity. Due to the increasing elderly population of the United States, it is imperative we identify the factors driving these disparities and address them. Our research provides a concrete starting point for future studies and potential policy changes to address these disparities in access to care.

## Appendices

Script

“Hi, I am calling on behalf of my grandfather. He has been experiencing lower back pain for quite some time now. He was seen by his Primary Care Doctor; however, he continues to have pain. Does Dr. Jones treat lower back pain?” If answered yes, “Is your office accepting new patients at this time?” If answered yes, “My grandfather has Medicare/Humana Gold Plus HMO/ Blue Medicare Select PPO/ Cigna, are you currently accepting that insurance?” If answered yes, “When is your next available appointment?” Prior to scheduling the appointment with the office staff, “Let me call my grandfather and see if that date and time works for him, thank you so much for your help.”

If asked for a referral prior to receiving the date and time of the next available appointment, “I will have his Primary Doctor send a referral to the office, but to plan, when is the next available appointment so I can check my grandfather's availability.” If asked about previous imaging, the caller was instructed to tell the clinic only X-rays have been taken.
